# A computational model of stem cells’ decision-making mechanism to maintain tissue homeostasis and organization in the presence of stochasticity

**DOI:** 10.1038/s41598-022-12717-0

**Published:** 2022-06-02

**Authors:** Najme Khorasani, Mehdi Sadeghi

**Affiliations:** 1 School of Biological Sciences, Institute for Research in Fundamental Sciences (IPM), Tehran, Iran; 2 National Institute of Genetic Engineering and Biotechnology (NIGEB), Tehran, Iran

**Keywords:** Cell biology, Computational biology and bioinformatics, Stem cells

## Abstract

The maintenance of multi-cellular developed tissue depends on the proper cell production rate to replace the cells destroyed by the programmed process of cell death. The stem cell is the main source of producing cells in a developed normal tissue. It makes the stem cell the lead role in the scene of a fully formed developed tissue to fulfill its proper functionality. By focusing on the impact of stochasticity, here, we propose a computational model to reveal the internal mechanism of a stem cell, which generates the right proportion of different types of specialized cells, distribute them into their right position, and in the presence of intercellular reactions, maintain the organized structure in a homeostatic state. The result demonstrates that the spatial pattern could be harassed by the population geometries. Besides, it clearly shows that our model with progenitor cells able to recover the stem cell presence could retrieve the initial pattern appropriately in the case of injury. One of the fascinating outcomes of this project is demonstrating the contradictory roles of stochasticity. It breaks the proper boundaries of the initial spatial pattern in the population. While, on the flip side of the coin, it is the exact factor that provides the demanded non-genetic diversity in the tissue. The remarkable characteristic of the introduced model as the stem cells’ internal mechanism is that it could control the overall behavior of the population without need for any external factors.

## Introduction

One of the major transitions in evolution is the major step from mere unicellularity into the new world of multicellularity. To understand this, one has to study two main characteristics of multicellular organisms that appeared during this transition, namely differentiation, and self-organization. These two processes are so tied up during development. To be more specific, through the self-organization process, genetically homogeneous differentiated groups of cells are organized into the tissues with different shapes and functionalities that can all together work as an overall patterned structure. For the proper functionality of the fully formed organism in a developed normal tissue, it is vital to have a strategy to generate the right proportion of different types of specialized cells, distribute them into their right position, and maintain the organized structure in a homeostatic state.

In a developed tissue, stem cells are the main source of producing cells^1^. Therefore the main focus of this project is understanding the stem cells’ internal mechanism which controls their decisions. The decisions which satisfy the primary goal of life: keeping the living organisms alive. Stem cells are characterized by their capacity to self-renew and differentiate into more specialized cell types^2,3^. Any imbalance between the tissue demand and these two can lead to dysfunctional tissues or tumorigenesis. Hence, here we introduce a computational model to understand the underlying mechanisms that orchestrate the stem cell proliferation/differentiation balance to regulate the non-genetic diversity, as well as maintain the spatial pattern in the dynamic stochastic environment of a living tissue^4^. This will be a step toward the big picture of our final project: introducing a comprehensive model to understand the stem cells’ internal activities regulating their final fate. That helps us study mysterious phenomena such as aging, and cancer, and manufacture artificial organs and develop stem-cell-based therapies in regenerative medicine.

That is for sure that, a non-genetic diversifying factor is required to produce hundreds of different cell types, in an organized pattern, from genetically similar stem cells. In this circumstance, stochasticity is advantageous and even critical^5–9^. Because of the low number of reacting molecules, a cell as a biochemical system is intrinsically probabilistic. Therefore, stochasticity is an inevitable part of a living cell, and it makes stochasticity an available suitable factor for cell-cell variability. An increasing number of theoretical and experimental studies unravel that the controlled amount of stochasticity could lead to phenotypic variations^10,11^ and population heterogeneity^5,6,12,13^, while maintaining a precise and robust regulation of key reactions in the cell. For this reason, stochasticity is observable in all the levels of our model. Needless to say, any perturbation from the optimum amount of stochasticity consequently diminishes the phenotypic diversity, and proliferation/differentiation balance.

The absence of the phenotypic diversity and proliferation/differentiation balance could doubtlessly cause severe damage to the structure of the tissue, which in the first place has been organized to provide its specific functionality. To prevent this, a robustly regulated stochastic decision-maker is required to maintain first, proliferation/differentiation balance to avoid tissue depletion or abnormal growth^3,14,15^, second, a non-genetic diversity which is critical to the survival of living systems in noisy environments^16–21^. On the other hand, cellular regulatory networks have been studied in a wide range of organisms and are known to control decision making from viruses^22–24^ to bacteria^25–28^, yeast^29^, and human embryonic stem cells^30–34^. Considering these facts, and inspired by previous studies that revealed the impact of regulatory networks on the stability of biological systems^6,10,23,29,35–39^, in our model, we introduce multi-stable regulatory networks as the internal decision-makers of dividing cells. Here, a set of ordinary differential equations (ODEs) are defined as the typical mathematical tool to study, and describe the regulatory circuitries^7,32,40^.

The regulatory networks could provide proliferation/differentiation balance and population heterogeneity which are sufficient to maintain homeostasis state in the population of cells with desired proportions of different phenotypes. However, these two issues are not adequate to conserve the population’s structural organization. Intercellular communication is responsible for instructing different cell types to transition from population heterogeneity in a homeostasis state to ordered spatial patterns a multi-cellular organism^13,41,42^. Therefore, it is necessary to equip the model with intercellular communication as the third issue required for surviving and functionality. In other words, it is assumed that the decision-maker in the stem cell in addition to being controlled by the intrinsic factors, being influenced by extracellular signals allowed to diffuse between the cells in the system^43^. To this purpose, a reaction–diffusion process is provided in the model in order to maintain a new steady-state with a spatial pattern.

Taking into account the probabilistic nature of any system with a small number of contributing determinants/chemicals associated with intra-/inter-cellular processes, it is vital to utilize a stochastic model for a better understanding of the system behavior. Gillespie algorithm is proven to be suitable for describing the behavior of the systems with a small number of determinants/chemicals driven by inherent fluctuations^6,44^. Besides, without having to deal with complex mathematical solutions, just averaging over enough number of simulations of the Gillespie algorithm could provide an asymptotic approximation to the exact numerical solution of the corresponding master equation. Therefore, to simulate the time evolution of our inherently stochastic system, here, the Gillespie algorithm is used.

Considering all the facts discussed before and focusing on the impact of stochasticity, here, we propose a computational model to reveal the mechanism which maintains the proliferation/differentiation balance as well as the spatial pattern in a hypothetical normal adult tissue. The proposed model is defined based on six material principles discussed in^7,13^ and reconsidered as follows: (1) stochasticity is an inevitable part of any living cell^5,7,13,38,45–49^. (2) Two major sources of stochasticity, the non-deterministic position of the cell division plane and nonuniform distribution of determinants in the cell lead to the random distribution of the cytoplasmic molecules among daughter cells during cell division^7,13,50–56^. (3) Cell fate is determined based on the number of determinants in the offspring upon the completion of cell division and assumed to be fixed during cell life cycle^7,13^. (4) Cell determinants interact with each other via an internal switch^16,57^. (5) The decision bias in the internal switch is determined by model parameters representing interactions between the switch elements^7,13^. (6) The switch parameters could also be affected by the cell location in its environment, and it is the key to the spatial pattern in the population^13^.

Studying spatial patterns in living organisms is challenging and limited to a few model organisms that can be easily imaged and manipulated. Besides, experimental methods are usually very expensive and face barriers that are so difficult to overcome. On the other hand, proposed computational methods study self-organization in particular cell types by considering a few specific factors. In this project, we propose a comprehensive model relying on stochasticity to describe self-organization in all cell types, based on signal diffusion in cell-cell communication. The overall outcome of our model implies that in the presence of controlled noise in a population of genetically similar cells, we could maintain population heterogeneity, homeostasis, and also structural pattern. Furthermore, we investigate how affecting the parameters in cell regulatory switches by the stochastic environmental factors could manipulate cellular decision-making bias and let the cells know their location in the population and their neighboring cells and maintain the initial organization as a result. We show that how the spatial order of cells in the population could be harnessed by the colony geometries. In addition, our model could easily retrieve its initial pattern in terms of injuries. Finally, to further illustrate the strength of the model, we explore the behavior of the system in the face of more complex structural patterns.

## Methods

### Mathematical model of the system

We consider a model for an adult normal tissue consisting of a population of stem cells (*S*) as the source of producing demanding cells and specialized (differentiated) cells to fulfill the tissue functionality. In most living tissues there is an intermediate population of cells between stem cells and terminally differentiated cells. These cells with a limited capacity of proliferation and restricted potential of differentiation, known as progenitor cells^58^. The primary role of progenitor cells is to produce an enormous number of specialized cells demanded in the tissue by going through many rounds of cell division^58^. Therefore, the production of thousands of differentiated cells could be maintained with a low rate of stem-cell division rate. This is beneficial in the sense that the larger number of stem cell division cycles, the greater is the risk of stem-cell mutations. This way, the presence of progenitor cells can prevent the accumulation of mutations which is sufficient to cause cancer. Therefore, it is assumed that the developing tissue that we are studying is made up of progenitor cells (*P*) as well as stem cells, and differentiated cell type^58,59^.

In our model, the stem cells can self-renew and also can be divided into progenitor cells which present a bi-stable system. Progenitor cells can give rise to only one or a few types of specialized non-dividing cells. Without loss of generality, here the progenitor cells can divide into two non-dividing differentiated cells termed *A*, and *B*, which die after several days or weeks^59^. From the dynamical system point of view in our model, the progenitor cells are studied as a tristable system, which is also biologically reliable.

Investigations of tissue regeneration unveil a remarkable degree of flexibility, with progenitor cells able to recover the stem cell presence following injury, and even under normal conditions. It is also has been shown that reversible transfer of cells provides a more viable mechanism to maintain homeostasis under all sort of conditions^60–70^. That being the case, in this model stem and progenitor cells can switch stochastically between two states: stem cells could transit to progenitor cells and vice versa with a fixed rate. In addition, it is assumed that stem and progenitor cells diminish with rate of $\gamma _S$, and $\gamma _P$. Within the described framework, the model dynamics could be described as follows:

1 $$\begin{aligned}&{S \xrightarrow {\eta } S + P}, \; {S \xrightarrow {\eta _S} S + S}, \; {S \xrightarrow {\eta _P} P + P}. \end{aligned}$$

2 $$ S\underset{{w_{S} }}{\overset{{w_{P} }}{\rightleftharpoons}}P.{\text{ }} $$

3 $$\begin{aligned}&{S \xrightarrow {\gamma _S} \Phi }, \; {P \xrightarrow {\gamma _P} \Phi } \end{aligned}$$

4 $$\begin{aligned}{}&\begin{aligned} {P \xrightarrow {\lambda _P} P + P}, \; {P \xrightarrow {\lambda _A} A + P}, \; {P \xrightarrow {\lambda _B} B + P}, \\ {P \xrightarrow {\mu _{d}} A + B}, \; {P \xrightarrow {\mu _{A}} A + A}, \; {P \xrightarrow {\mu _{B}} B + B}. \end{aligned} \end{aligned}$$

5 $$\begin{aligned}{}&{A \xrightarrow {\gamma _A} \Phi }, \; {B \xrightarrow {\gamma _B} \Phi } \end{aligned}$$

where $\eta$, $\eta _s$, and $\eta _p$ denote the rates of stem cell’s three different division types, $\omega _{p,s}$ represents the transition rates from *S* to *P*, and *S* to *P*, respectively, and $\lambda _p$, $\lambda _A$, $\lambda _B$, $\mu _d$, $\mu _A$, and $\mu _B$ denote the rates of progenitor cell’s six different division types. The last two processes denote the rates, $\gamma _A$, and $\gamma _B$, at which *A*, and *B* cells commit to death.

The time evolution of the average densities of cell type *S*, cell type *P*, cell type *A*, and of cell type *B*-where $n_S$, $n_P$, $n_A$, and $n_{B}$ are cell numbers normalized by volume-is given by

6 $$\begin{aligned} \left\{ \begin{aligned} \partial _t n_S&= f(n_S,n_P,n_A,n_B) \\&= n_S \eta _S - n_S \eta _P+n_P w_S -n_S w_P-n_S\gamma _S, \\ \partial _t n_P&=g(n_S,n_P,n_A,n_B)\\&= n_S\eta +2n_S\eta _P+n_S w_P-n_P w_S-n_P\gamma _P \\&-n_P(-\lambda _P+\mu _d+\mu _A+\mu _B), \\ \partial _t n_A&=h(n_S,n_P,n_A,n_B)\\&= n_P(\lambda _A+\mu _d+2\mu _A)-n_A\gamma _A, \\ \partial _t n_B&=q(n_S,n_P,n_A,n_B)\\&= n_P(\lambda _B+\mu _d+2\mu _B)-n_B\gamma _B. \end{aligned} \right. \end{aligned}$$

In steady state:

7 $$\begin{aligned} \begin{aligned}{}&\partial _t n_S = 0\\&\partial _t n_P = 0\\&\partial _t n_A = 0 \\&\partial _t n_B = 0, \end{aligned} \end{aligned}$$

then,

8 $$\begin{aligned} \begin{aligned}{}&n_S^* = n_P^*\frac{-w_S}{\eta _S-\eta _P-\gamma _S-w_P}\\&w_S^* = \frac{(\eta _S-\eta _P-w_P-\gamma _S)(-\gamma _P+\lambda _P-\mu _d-\mu _A-\mu _B)}{\eta +\eta _P+\eta _S-\gamma _S},\\&n_A^* = n_P(\lambda _A+\lambda _d+2\mu _A)/\gamma _A,\\&n_B^* = n_P(\lambda _B+\lambda _d+2\mu _B)/\gamma _B. \end{aligned} \end{aligned}$$

As $n_S^*>0$, $n_P^*>0$, and $w_S>0$ then:

9 $$\begin{aligned} \begin{aligned}{}&\eta _S-\eta _P-\gamma _S-w_P<0. \end{aligned} \end{aligned}$$

Besides, it is clear that $w_S^*>0$, then by considering Eq. (9), one of the following conditions should be satisfied:

10 $$\begin{aligned} \begin{aligned}{}&\eta +\eta _P+\eta _S<\gamma _S, \; and \; \lambda _P > \gamma _P+\mu _d+\mu _A+\mu _B, \end{aligned} \end{aligned}$$

or

11 $$\begin{aligned} \begin{aligned}{}&\eta +\eta _P+\eta _S>\gamma _S, \; and \; \lambda _P < \gamma _P+\mu _d+\mu _A+\mu _B. \end{aligned} \end{aligned}$$

Progenitor cells are known as the intermediate type of cells with a limited capacity of proliferation^58^. In other words, after a limited number of cell divisions, they terminally differentiate to fulfill their responsibility in producing specialized cells in a normal mature tissue^59^. Consequently, it is interpreted that the progenitor cells’ potential to self-renew lowered through generations. In our model, it is made to happen by setting the parameters in a way that the conditions in Eq. (11) are satisfied. Because, in contrast to the second condition in Eq. (10), when the conditions in Eq. (11) are satisfied, through generations it becomes more and more probable for a progenitor cell to be differentiated than self-renew. It can be easily interpreted as the limited capacity of proliferation in progenitor cells.

To study the stability of the model, it is needed to compute the Jacobian matrix of Eq. (6):

12 $$\begin{aligned} J(n_S,n_P,n_A,n_B)= \begin{pmatrix} \partial _{n_S}F&{}\partial _{n_P}F&{}\partial _{n_A}F&{}\partial _{n_B}F\\ \partial _{n_S}G&{}\partial _{n_P}G&{}\partial _{n_A}G&{}\partial _{n_B}G\\ \partial _{n_S}P&{}\partial _{n_P}H&{}\partial _{n_A}H&{}\partial _{n_B}H\\ \partial _{n_S}Q&{}\partial _{n_P}Q&{}\partial _{n_A}Q&{}\partial _{n_B}Q \end{pmatrix} \end{aligned}$$

where $f(n_S,n_P,n_A,n_B)$, $g(n_S,n_P,n_A,n_B)$, $h(n_S,n_P,n_A,n_B)$, and $q(n_S,n_P,n_A,n_B)$ are denoted as *F*, *G*, *H*, and *Q*.

The system fixed point, $(n_S^*,n^*_P,n_A^*,n_B^*)$, is stable if all four eigenvalues of *J*, or all the roots of equation $det(J-\lambda I)=0$, at the fixed point are negative^71^. For $\kappa = (\eta _S-\eta _P-w_P-\gamma _S)-\lambda$, $\theta =(\eta +2\eta _P+w_P)-\lambda$, $\xi =(-w_S-\gamma _P+\lambda _P-\mu _d-\mu _A-\mu _B)-\lambda$, $\chi =\lambda _A+\mu _d+2\mu _A$, $\upsilon =(-\gamma _A)-\lambda$, $\psi =\lambda _B+\mu _d+2\mu _B$, and $\phi =(-\gamma _B)-\lambda$, we get:

13 $$\begin{aligned} J-\lambda I= \begin{pmatrix} \kappa &{}w_S&{}0&{}0\\ \theta &{}\xi &{}0&{}0\\ 0&{}\chi &{}\upsilon &{}0\\ 0&{}\psi &{}0&{}\phi \end{pmatrix}. \end{aligned}$$

In this case, $det(J-\lambda I)= (\kappa \xi -w_s\theta )\upsilon \phi$ which is independent from the values of $\chi$, and $\psi$. Therefore, one could say that from the stability point of view, our system in (6) is equivalent to two independent two-dimensional systems as follows:

14 $$ \left\{ \begin{aligned}   \partial _{t} n_{S}  & = n_{S} \eta _{S}  - n_{S} \eta _{P}  + n_{P} w_{S}  - n_{S} w_{P}  - n_{S} \gamma _{S} , \\    \partial _{t} n_{S}  & = n_{S} \eta _{S}  - n_{S} \eta _{P}  + n_{P} w_{S}  - n_{S} w_{P}  - n_{S} \gamma _{S} , \\     & \quad  - n_{P} ( - \lambda _{P}  + \mu _{d}  + \mu _{A}  + \mu _{B} ),   \end{aligned}  \right. $$

and

15 $$\begin{aligned} \left\{ \begin{aligned} \partial _t n_A = n_P(\lambda _A+\mu _d+2\mu _A)-n_A\gamma _A, \qquad \quad \quad \qquad \quad \, \\ \partial _t n_B = n_P(\lambda _B+\mu _d+2\mu _B)-n_B\gamma _B.\quad \quad \quad \qquad \qquad \,\,\, \end{aligned}\right. \end{aligned}$$

In the former two-dimensional system, $det(J'-\lambda I)= \kappa \xi -w_s\theta$ (first term in $det(J-\lambda I$)), and in the latter one, $det(J''-\lambda I)=\upsilon \phi$ (second term in $det(J-\lambda I$)). Hence, if all four roots of these two equations are proven to be negative, the condition for the stability of the system in Eq. (6) is fulfilled.

Here, we aim to study the conditions which lead to the stability of the first system’s fixed point, namely, $(n_s^*, n_p^*)$. The time evolution of the average densities of cell type *S*, $n_s$, cell type *P*, $n_P$ is given in Eq. (14). Clearly, the average total density of dividing cells (stem and progenitor cells), *n*, could be computed as follows:

16 $$\begin{aligned} n = n_S+n_P, \end{aligned}$$

then,

17 $$\begin{aligned} \begin{aligned} \partial _t n &= n_S(\eta _S+\eta _P+\eta -\gamma _S)\\&\quad +n_P(-\gamma _P+\lambda _P-\mu _A-\mu _B-\mu _d-\eta _S-\eta _P-\eta +\gamma _S). \end{aligned} \end{aligned}$$

When we substitute $n_S=n-n_P$, the Eq. (14) becomes:

18 $$\begin{aligned} \begin{aligned} \partial _t n_P = f'(n_P,n)\\ \partial _t n \;\,= g'(n_P,n), \end{aligned} \end{aligned}$$

where we introduced the functions

19 $$\begin{aligned} \begin{aligned} f'(n,n_P) &= n(\eta +2\eta _P+w_P)\\&\quad +n_P(-w_S-\gamma _P-w_P-\eta +\lambda _P-\mu _d-\mu _A-\mu _B-2\eta _P)\\ g'(n,n_P) &= n(\eta _S+\eta _P+\eta -\gamma _S)\\&\quad +n_P(-\gamma _P+\lambda _P-\mu _A-\mu _B-\mu _d-\eta _S-\eta _P-\eta +\gamma _S)\\ \end{aligned} \end{aligned}$$

To study the stability of the model, we compute the Jacobian matrix of Eq. (18),

20 $$\begin{aligned} J'(n_P,n)= \begin{pmatrix} \partial _{n_P}F'&{}\partial _{n}F'\\ \partial _{n_P}G'&{}\partial _{n}G' \end{pmatrix} \end{aligned}$$

where, $f'(n_P,n)$, and $g'(n_P,n)$ are denoted as $F'$, and $G'$.

The fixed point $(n_P^*,n^*)$ is stable if both eigenvalues of $J'$ at the fixed point are negative. This is the case if

21 $$\begin{aligned} det(J'(n_P^*,n^*))>0, \; and \;tr(J'(n_P^*,n^*))<0. \end{aligned}$$

For the system defined in Eqs. (18), and (19), the Jacobian reads as Eq. (25), where $\zeta = -w_S-\gamma _P-w_P-\eta +\lambda _P-\mu _d-\mu _A-\mu _B-2\eta _P$, $\,\vartheta = -\gamma _P+\lambda _P-\mu _A-\mu _B-\mu _d-\eta _S-\eta _P-\eta +\gamma _S$, and $\eta _S=\eta _S(n)$.

22 $$\begin{aligned} J'(n_P,n)= \begin{pmatrix} \zeta &{}\eta +2\eta _P+w_P\\ \vartheta &{}\eta _S+\eta _P+\eta -\gamma _S+n\eta _S'(n) \end{pmatrix} \end{aligned}$$

In steady state we could substitue $w_S = w_S^*$ (Eq. 8), and compute $det(J'(n_P, n))$, and $tr(J'(n_P, n))$ as follows:

23 $$\begin{aligned}{}&\begin{aligned} det(J'(n_P, n))&=\zeta * [\eta _S+\eta _P+\eta -\gamma _S+n\eta _S'(n)]\\&-\vartheta *[\eta +2\eta _P+w_P]\\&= \zeta n\eta _S'(n) \end{aligned} \end{aligned}$$

24 $$\begin{aligned}{}&\begin{aligned} tr(J'(n_P,n)&=\zeta +\eta _S+\eta _P+\eta -\gamma _S+n\eta _S'(n)\\&=(-w^*_S)+(\eta _S-\eta _P-\gamma _S-w_P)+(\lambda _P-\gamma _P-\mu _d-\mu _A-\mu _B) \end{aligned} \end{aligned}$$

It is clear that $det(J'(n_P,n))>0$, and $tr(J'(n_P,n))<0$ as long as $\eta _S'(n)<0$, and the conditions in Eqs. (9), and 11 are satisfied. Therefore, the fixed point $(n_P^*,n^*)$ is stable.

The Jacobian matrix of the second system (Eq. 15) reads:

25 $$\begin{aligned} J''(n_A,n_B)= \begin{pmatrix} -\gamma _A&{}0\\ 0&{}-\gamma _B \end{pmatrix} \end{aligned}$$

In this system, as $det(J''(n_A,n_B))=\gamma _A\gamma _B>0$, and $tr(J'-(n_A,n_B)) = -\gamma _A-\gamma _B<0$, the fixed point, ($n_A^*$, $n_B^*$) is stable. Therefore, it can be instantly concluded that the system fixed point of $(n_S^*, n_P^*, n_A^*, n_B^*)$ is stable.

### Stem cells’ internal mechanism

The regulatory mechanism which provides the first part of the model dynamics in (1) is described by a set of ordinary differential equations (ODEs) which previously was used in several studies^7,32,40^. The following set of ODEs are employed to describe the stem cells’ internal mechanism as a two-element bi-stable regulatory switch:

26 $$\begin{aligned} \left\{ \begin{aligned} \frac{dx_s}{dt}=\iota _{x_s} \frac{\beta _s^n}{\beta _s^n+y_s^n}-\gamma x_s \\ \\ \frac{dy_s}{dt}=\iota _{y_s} \frac{\beta _s^n}{\beta _s^n+x_s^n}-\gamma y_s \\ \end{aligned}\right. \end{aligned}$$

**Figure 1:**
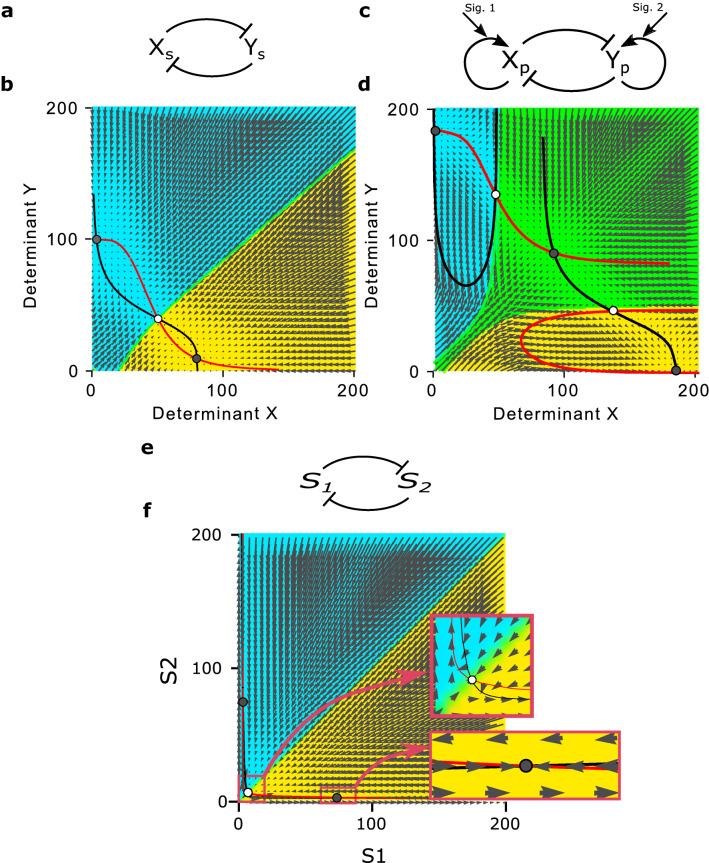
The model’s regulatory networks together with their corresponding force-field representations. The nullclines are drawn in red and black (**a**) A bi-stable regulatory network. (**b**) The stem cell’s force-field representation. (**c**) A tristable regulatory network. (**d**) The progenitor cell’s force-field representation. (**e**) The signalling molecules’ bi-stable regulatory network. (**f**) The signalling molecules’ force-field representation. Two critical zones around two attractors in the field are zoomed in in two red frames.

In this model, It is assumed that the cell type is controlled by the relative amount of two cytoplasmic cell fate determinants, namely $X_s$ and $Y_s$ whose interactions can be described in a form of a bi-stable regulatory switch (see Fig. 1a). The dynamical behavior of the determinants $X_s$ and $Y_s$ is studied by considering their mutual repression effect which is modeled in the form of a Hill function^7,13,23^, and their degradation rate. Here, *n* is the Hill coefficient, $\beta _s$ is the effective rate of determinants synthesis, $\iota _{X_s}$ and $\iota _{Y_s}$ are inhibition rates, and $\gamma$ is the degradation rate.

The parameters of Eq. (26) are set in such a way that there will be two stable steady states, as shown in Fig. 1b, corresponding to two different cell fates, stem cell type *S* and progenitor cell types *P*. The number of determinants of $X_s$ ($Y_s$) involved in attractor *S* (*P*) is much larger than those of $Y_s$ ($X_s$). Figure 1b represents the domains of the two attractors, *S*, and *P*, with two different colors, yeloow and blue, respectively. Each daughter cell with a specific value of $X_s$ and $Y_s$, right after birth, can be shown as a point in Fig. 1b. The value of $X_s$ and $Y_s$ determines to which attractor the cell will be absorbed, and based on that it defines the domains of two attractors. In other words, each cell fate can be determined and fixed exactly after division based on the number of determinants $X_s$ and $Y_s$ in the daughter cell.

Since, based on Eq. (26), $\iota _{x_s}$, and $\iota _{y_s}$ are the parameters which determine the number of determinants of $X_s$ ($Y_s$) involved in stem cells, it can be easily concluded that they are the parameters which control the rate of symmetric divisions ($S \xrightarrow {\eta _S} S + S$, and $S \xrightarrow {\eta _P} P + P$) directly. Furthermore, to have the first condition in (11) be satisfied in steady state, we set $\eta _S<\eta _P$. Therefore, for this purpose it is enough to set $\iota _{x_s}<\iota _{y_s}$. Besides, corresponding to $\eta _S=\eta _s(n)$, in our model $\iota _{x_s}$ is considered as a function of *n*, in a way that $\iota _{x_s}(n)'<0$.

### Progenitor cells’ internal mechanism

The regulatory mechanism of the progenitor cells (shown in Fig. 1c) is described by the following set of ordinary differential equations (ODEs) as a two-element tristable system^7,32,40^:

27 $$\begin{aligned} \left\{ \begin{aligned} \frac{dx_p}{dt}=(\alpha _{x_p}+\varepsilon _{s_1})\frac{x_p^n}{\beta _p^n+x_p^n}+\iota _{x_p} \frac{\beta _p^n}{\beta _p^n+y_p^n}-\gamma x_p \\ \\ \frac{dy_p}{dt}=(\alpha _{y_p}+\varepsilon _{s_2})\frac{y_p^n}{\beta _p^n+y_p^n}+\iota _{y_p} \frac{\beta _p^n}{\beta _p^n+x_p^n}-\gamma y_p \\ \end{aligned}\right. \end{aligned}$$

Likewise, It is assumed that the cell’s final fate is determined by the relative amount of two cytoplasmic determinants, namely $X_p$ and $Y_p$ whose interactions can be described in a form of a tristable switch (see Fig. 1c). The dynamical behavior of the determinants $X_p$ and $Y_p$ is studied by considering their self-activation, and mutual repression effects which are modeled in the form of a Hill function^7,13,23^, and their degradation rate. In the same manner as the bi-stable system, *n*, $\beta _p$, $\iota _{X_s/Y_s}$, and $\gamma$ plays their role as the Hill coefficient, the effective rate of determinants synthesis, inhibition, and degradation rate, respectively. Besides, there are four more parameters, $\alpha _{x_p}$, and $\alpha _{y_p}$, as activation rates, and $\varepsilon _{s_1}$, and $\varepsilon _{s_2}$ as “signalling effect coefficients” (will be discussed in this session).

As shown in Fig. 1d, the parameters of Eq. (27) lead to a tristable steady-state system. In this system, there are three fixed points corresponding to three different cell fates, one progenitor cell type, *P*, and two differentiated cell types, namely, *A*, and *B*. The number of determinants $X_p$, and $Y_p$ are in balance in *P* cells, where in *A* (*B*) cells the number of determinants $X_p$ ($Y_p$) exceeds that of determinants $Y_p$ ($X_p$). Figure 1d represents the domains of the three attractors, corresponding to *P*, *A*, and *B* cells, with three different colors, green, yellow, and blue respectively. As the number of determinants in the cell is updating, their corresponding trajectory in the phase plane is changing and finally reaches the domain of their attractor. It is worth mentioning that each cell fate can be finalized right after its birth based on the number of determinants $X_p$ and $Y_p$ in it.

**Figure 2:**
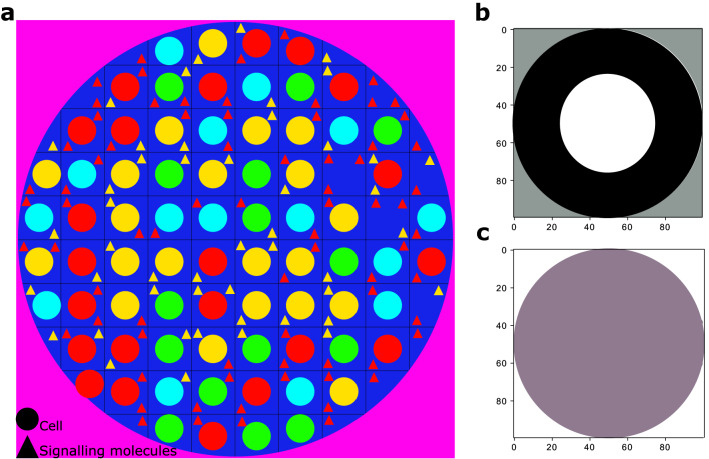
Hypothetical dish as a main scene for our system dynamics, and the filters representation for scoring algorithm. (**a**) the hypothetical dish is occupied with four types of cells, *S* (cyan), *P* (green), *A* (yellow), and *B* (red). Yellow, and red triangles represent signalling molecules namely, $S_1$, and $S_2$, produced by cell types *A* and *B*, respectively. (**b**,**c**) Filters representing template, and penalty matrices corresponding to the perfect borders of the population initial pattern and the valid territory of the entire dish, respectively.

Representing in Fig. 2a, it is assumed that there is a dish, occupied with four types of cells, *S* (cyan), *P* (green), *A* (yellow), and *B* (red), as the main scene for our system dynamics. As another assumption in our model, cell types *A*, and *B* produce signaling molecules namely, $S_1$ (yellow triangles), and $S_2$ (red triangles), respectively. Depicted in Eq. (27), theses signalling molecules could affect the self-activation rate of the progenitor cells through the parameters $\varepsilon _{s_1}$, and $\varepsilon _{s_2}$. The signaling effect coefficients read as follows:

28 $$\begin{aligned} \varepsilon _{s_i}= \left\{ \begin{aligned} \frac{a S_i}{b} \;\;\; if\;\; S_i\le b\\ \\ a \;\;\;\;\;o.w. \\ \end{aligned}\right. \end{aligned}$$

where $i \in \{1,2\}$, $a=10$, and $b=15$. Clearly, when in a part of dish, number of signalling molecules $S_1$ ($S_2$) increases, $\varepsilon _{s_1}$ ($\varepsilon _{s_2}$) increases in value. As a result, the self-activation effect of $x_p$ ($y_p$) on itself grows, and the birth of *A* (*B*) daughter cells in that part of dish will be more probable. In the other words, by increasing the number of $S_1$ ($S_2$) molecules, *A* (*B*) cells conquer the territory from other types of cell. Another point to mention is that *a*, and *b* values are set in such a way that territory of *A* (*B*) cells in dish remains unchanged.

As it is studied before^7^, $\beta _p$ is the parameter which determines the position of two boundaries in the force-field plane (in Fig. 1d). In other words, by shrinking (expanding) the progenitor cell’s territory (the green domain), it controls the proportion of daughter cells which remain as progenitor cells, or differentiate to specialized cells. It can be easily concluded that it can directly control the rate of different types of divisions. To be specific, in our model, $\beta _p$ is set in such a way that the rate of symmetric division, $P \xrightarrow {\lambda _P} P + P$ is much less than divisions in the forms of: $P \xrightarrow {\mu _d} A + B$, $P \xrightarrow {\mu _A} A + A$, and $P \xrightarrow {\mu _B} B + B$. Therefore, the second condition in (11) will be satisfied in a steady state. To sum up, for each progenitor cell four reactions, *X*/*Y* synthesis/degradation, and one division process could occur, at each time step.

### Signalling dynamics

As discussed in “Progenitor cells’ internal mechanism”, it is assumed that differentiated cells, *A*, and *B* secrete signaling molecules, $S_1$, and $S_2$ respectively. The diffusion of signaling molecules between the pixels of the dish (Fig. 2a) can be governed by a set of reaction-diffusion equations as follows:

29 $$\begin{aligned} \left\{ \begin{aligned} \frac{ds_1}{dt}=D \nabla ^2s_1+\alpha _{s_1} \frac{\beta ^n}{\beta ^n+s_2^n}-k s_1 \\ \\ \frac{ds_2}{dt}=D \nabla ^2s_2+\alpha _{s_2} \frac{\beta ^n}{\beta ^n+s_1^n}-k s_2 \\ \end{aligned}\right. \end{aligned}$$

Here *D*, and, *k* represent, respectively, diffusion coefficient and the rate of decay of the signaling molecules, and $\alpha _{s_1}$ ($\alpha _{s_2}$) is the production rate. The number of signaling molecules at each point of the dish is shown by terms, $s_1$, and $s_2$, and their interactions are described in a form of a bi-stable regulatory switch (see Fig. 1e). The second term in Eq. (29) demonstrates the mutual inhibition effect of the signaling molecules, $S_1$, and $S_2$ in the form of a Hill function. Here, *n* is the Hill coefficient, $\beta$ is the effective rate of signaling molecules synthesis.

Figure 1f represents the domains of the two attractors corresponding to $S_1$, and $S_2$, with two different colors, blue, and yellow, respectively. Each pixel of the dish with a specific value of $S_1$, and $S_2$ can be shown as a point in Fig. 1f. The values of $S_1$, and $S_2$ determines which attractor the cell will be absorbed to, and based on that it defines the domains of two attractors. Therefore, in the steady state, and in the deterministic environment, each pixel could only contain $S_1$ or $S_2$, and not both.

In the simulations, the production of signaling molecules $S_1$ ($S_2$) is proportional to the number of *A* (*B*) cells. When differentiated cells emerge in the dish, their corresponding signaling molecules diffuse in their environment where they interact with each other based on Eq. (29). The number of signaling molecules at any location in the dish determines how much a progenitor cell at that location is affected by the signal values. As shown in Eq. (27), the number $S_1$ ($S_2$) will increase the birth rate of *A* (*B*) cells. Therefore, it is expected that if some parts of the dish are occupied by *A* (*B*) cells, it remains the same.

### Gillespie algorithm

The time evolution of the system is captured by the Gillespie algorithm^7,13,44^ which is known as the gold standard for simulating models whose stochasticity arises from the small discrete number of reactant molecules^72^. In each time step 24 different reactions can potentially happen. Table 1 demonstrates all the reactions and their corresponding propensity functions. In each iteration, one of the above-mentioned processes occurs, time is updated, and the simulation continues to the point that all progenitor cells have gone through at least 50 divisions.

**Table 1:** The potential reactions of the system together with their corresponding propensity functions

No	The reactions	The Propensity Func.	No	The reactions	The Propensity Func.
1	Production of $X_s$	$\iota _{x_s} \frac{\beta _s^n}{\beta _s^n+y_s^n}$ $\iota _{x_s} = 85, \beta _s = 45, n = 4$	12	Degredation of $Y_p$	$\gamma y_p \gamma =0.38$
2	Degredation of $X_s$	$\gamma x_s \gamma =1$	13	Prg. Cell Devision	$r_p r_p = 32.9$
3	Production of $Y_s$	$\iota _{y_s} \frac{\beta _s^n}{\beta _s^n+x_s^n}$ $\iota _{y_s}=100,\beta _s = 45,n=4$	14	Prg. Cell Death	$\gamma _p$ $\gamma _p= 0.01504$
4	Degredation of $Y_s$	$\gamma y_s$ $\gamma =1$	15	Prg. Cell Transformation	$w_s$ $w_s=0.1645$
5	SC. Division	$r_s$ $r_s=56.4$	16	Prg. Cell Movement	$m_p$ $m_p=0.94$
6	SC. Death	$\gamma _s$ $\gamma _s=0.0423$	17, 18	*A*/*B* cell death	$\gamma _{A/B}$ $\gamma _{A}= 0.003, \gamma _{B}=0.0034$
7	SC. Transformation	$w_p$ $w_p=0.329$	19	Production of $S_{1}$	$\alpha _{s_{1}} \frac{\beta ^n}{\beta ^n+s_{2}^n}$ $\alpha _{s_{1}}=220,\beta =2,n=4$
8	SC. Movement	$m_s$ $m_s=2.82$	20	Degradation of $S_{1}$	$k s_{1}$ $k =0.5$
9	Production of $X_p$	$(\alpha _{x_p}+\varepsilon _{s_1})\frac{x_p^n}{\beta _p^n+x_p^n}+\iota _{x_p} \frac{\beta _p^n}{\beta _p^n+y_p^n}$ $\alpha _{x_p}=30,\iota _{x_p}=30,\beta _p=47.5,n=4$	21	Production of $S_{2}$	$\alpha _{s_{2}} \frac{\beta ^n}{\beta ^n+s_{1}^n}$ $\alpha _{s_{2}}=220,\beta =2,n=4$
10	Degredation of $X_p$	$\gamma x_p$ $\gamma =0.38$	22	Degradation of $S_{2}$	$k s_{2}$ $k=0.5$
11	Production of $Y_p$	$(\alpha _{y_p}+\varepsilon _{s_2})\frac{y_p^n}{\beta _p^n+y_p^n}+\iota _{y_p} \frac{\beta _p^n}{\beta _p^n+x_p^n}$ $\alpha _{y_p}=30,\iota _{y_p}=30,\beta _p=47.5,n=4$	23, 24	Diffusion of of $S_{1/2}$	$\frac{D}{h^2}$ $D=110,h=1$

The simulation starts with initializing the hypothetical dish (shown in Fig. 2a) with four types of cells and signalling molecules in an organized pattern. The number of determinants in stem (progenitor) cells, $X_S$, and $Y_S$ ($X_P$, and $Y_P$), are initialized randomly from the corresponding attractor territory. The number of signalling molecules in each mesh is chosen corresponding to the initial pattern in the population. The meshes with *A* (*B*) cells contains maximum number, almost 100, of $S_1$ ($S_2$) signalling molecules. In a mesh occupied with a stem (progenitor) cell, four reactions, production/degradation of determinant $X_S$ ($X_P$), and production/degradation of determinant $Y_S$ ($Y_P$) can potentially happen. In the deterministic manner, the ODE in Eq. (26) (Eq. 27) provides the exact description of these four reactions in our bistable (tristable) system. The propensity function of production/degradation reaction of determinant $X_S$ ($Y_S$) is determined based on the positive/negative term in the first (second) ODE in Eq. (26) (rows 1–4 in Table 1). The propensity functions of production/degradation reaction of determinants $X_P$, and $Y_P$ follow the same rule (see rows 9–12 captured based on Eq. (27)). At each time step, based on the probabilities corresponding to the propensity functions, the Gillespie algorithm determines which reaction occurs. As a result, in the current mesh, the determinants of the stem (progenitor) cell decrease or increase in number.

In a mesh with signalling molecules, based on Eq. (29), other than production/degradation of $S_1$, and $S_2$, diffusion is the fifth reaction to happen. The propensity functions for production/degredation of $S_1$ ($S_2$), are determined based on second/third term in the first (second) ODE in Eq. (29) (see rows 19–22 in Table 1). The propensity function of the diffusion process is equal to $D/h^2$ (rows 23, and 24 in Table 1). The production/degradation of signalling molecules occur in the system similar to the ones for dividing cells’ determinants. However, when diffusion is the selected candidate to occur in the system, one of the neighboring meshes of the current mesh is selected, and the number of signalling molecules in the current mesh is decreased while the number of that in the neighboring mesh increases. The neighbor with much more number of signalling molecules comparing to the current mesh is more probable to be selected for the diffusion process. It is worth noting that, the above-mentioned propensity functions representing high order reactions could be used only as an approximation with Gillespie algorithm^73^.

The propensity functions corresponding to the rest of reactions are chosen as constant rates in such a way that satisfies the conditions introduced in “Mathematical model of the system”. In the case of death, the cell in the current mesh is omitted from the population. In the case of division, one of the empty neighboring meshes is chosen for one of the newborn daughter cell. It is assumed that the distribution of determinants in each daughter cell is binomial^73^ with parameters specified according to the whole number of determinants in the mother cell, and probability of success for each trial, $p=1/2$ ($\sim B(\#X, 1/2)$, or $\sim B(\#Y,1/2)$, respectively).

All above-mentioned reactions are discussed before except the 8th, and 16th reactions, the stem, and progenitor cells’ movement. In a real medium, when a cell divides its offspring could push their neighboring cells to make some space, and there is always a movement in the dish. However, reflecting these actions is beyond our simplified model. In our model we need this movement otherwise, in the regions where progenitors divide to non-dividing cells, the system would be blocked. In this case, *A*, and *B* cells can border progenitor cells, and since there will be no space for newborn cells, it restricts the cell division. To prevent a blocking system and to avoid physics complications to study the cell movements, it is simply assumed that dividing cells can move in the dish at a fixed rate. In the simplest form of the movement process, a dividing cell can change its position to a randomly chosen empty position in the dish.

### Scoring algorithm

To evaluate the strength of our model in maintaining the population pattern, we define two filters corresponding to the initial state of the medium, shown in Fig. 2b,c. These filters representing two matrices called template (corresponding to Fig. 2b) and penalty (corresponding to Fig. 2c) matrices from now on. The element values of the template matrix are equal to 1 in the middle disk of the current dish, they are equal to $-1$ in the outer ring, and the rest of the values are equal to 0. The elements of the penalty matrix are equal to 1 out of the current dish and are equal to 0 otherwise.

Each simulation starts with an initial state with a desired spatial pattern and continues to the point that all progenitor cells have gone through 50 divisions on average. We select 500 $d\times d$ shots out of all the states model meets during the simulation, and produce 500 $2d\times 2d$ matrices corresponding to them. Each pixel in the medium representation could be mapped to an element in a $d \times d$ matrix. Next, we add *d*/2 *zeros* to each of the four sides of the matrices. In all of these 500 matrices, elements corresponding to yellow/red pixels are set to 1/$-1$, and the rest of the elements are set equal to 0. We slide the template, and penalty filters over each of the 500 matrices, multiply their corresponding values one by one and compute the summations. For each shot we do get two sets of $d+1$ values, namely $\{t_1, t_2,\dots ,t_{d+1}\}$, $\{p_1, p_2,\dots ,p_{d+1}\}$, corresponding to template and penalty filters, respectively. The assigned score value for each shot is calculated as follows:

$$\begin{aligned} \max _{i} (t_i-p_i); \;\;  i \in \{1,2,\dots ,d+1\}. \end{aligned}$$

Finally we normalize the score values in the range [0, 1].

## Results

### Maintaining the spatial pattern in the population

Aiming to study the capability of the model in maintaining the structural pattern of the population and in the presence of signalling molecules, the cells in the medium are initially organized in a circular pattern in which the dish is divided to two regions, a inner disk and an outer ring. It is assumed that the inner disk is mostly occupied with yellow *A* cells, where the outer ring is mainly filled by red *B* cells. Stem (presented in cyan) and progenitor (presented in green) cells, as dividing cells, are uniformly distributed in the dish.

**Figure 3:**
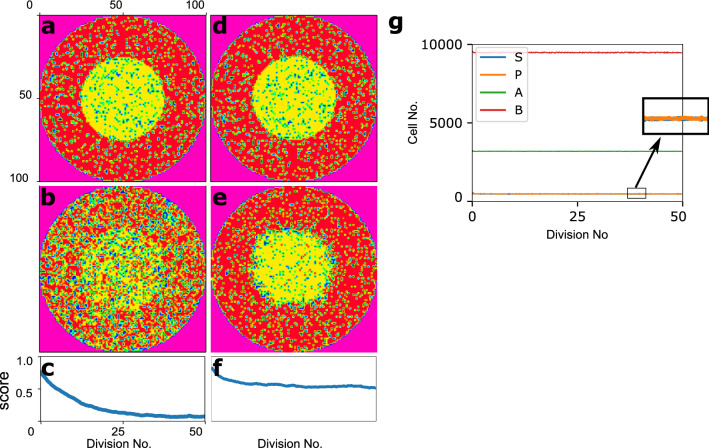
The system behaviour in the absence/presence of inter-cellulat interactions. (**a**,**d**) The initial state of the system. (**b**,**e**) The final states of the system in the absence/presence of signalling molecules, in row. (**c**,**f**) The corresponding score diagrams. (**g**) The maintenance of fours cell types’ abundance through time.

The first two simulations start with a population of *n* cells in a dish similar to the one presented in Fig. 2a. As shown in Fig. 3a, (and also Fig. 3d), the dish radius is initiated equal to 50 in the sense of placing at most 50 pixels on the dish radius. The inner disk radius is equal to 25. To compute the time evolution of the cell populations, a stochastic simulation, using Gillespie algorithm, is applied, and the simulation is terminated when the progenitor cells have gone through 50 divisions on average.

The first simulation is run in the absence of signaling molecules, and as expected the pattern could not be preserved in the population under this condition (Fig. 3b). However, in the second simulation, it is assumed that molecules *A*, and *B* produce signaling molecules as it was discussed before in “Signalling dynamics”. The final state is shown in Fig. 3e. Visually studying the first (Fig. 3d) and last (Fig. 3e) states of the system, as well as the abundance of four cell types through time (shown in Fig. 3g), it is clear that in the second simulation, both abundance of cell types, and the initial pattern are maintained properly.

To evaluate the results by numbers, 500 shots are selected out of all the states model meets during the simulation. As explained in “Scoring algorithm”, 500 corresponding scores are calculated and plotted in Fig. 3c,f, for the first and second simulations respectively. One could say that our scoring algorithm compares each of the 500 shots with the introduced filter in Fig. 2b. The filter which is chosen corresponding to the initial state of the system. In this case, diagrams in Fig. 3c,f demonstrates how much the population structural pattern differs from its initial pattern through the simulation. Figure 3c shows that at the end of the simulation the similarity between the initial and final states of our system is less than 10%. It clearly verifies that the initial pattern could not be maintained in the absence of intercellular interactions. Though Fig. 3f demonstrates the similarity score of $\simeq 60\%$ which indicates the model strength in maintaining the structural pattern in the presence of signaling molecules. Besides, in both cases, the score value diagram is saturated and it confirms the stability in the model which was promised in “Mathematical model of the system”. To put it another way, our system reaches the point that the structural pattern and the number of different cells will not change anymore. Though the stochasticity is observable in all levels of the system dynamic.

### Size of dish, and the inner disk affects the population spatial pattern

**Figure 4:**
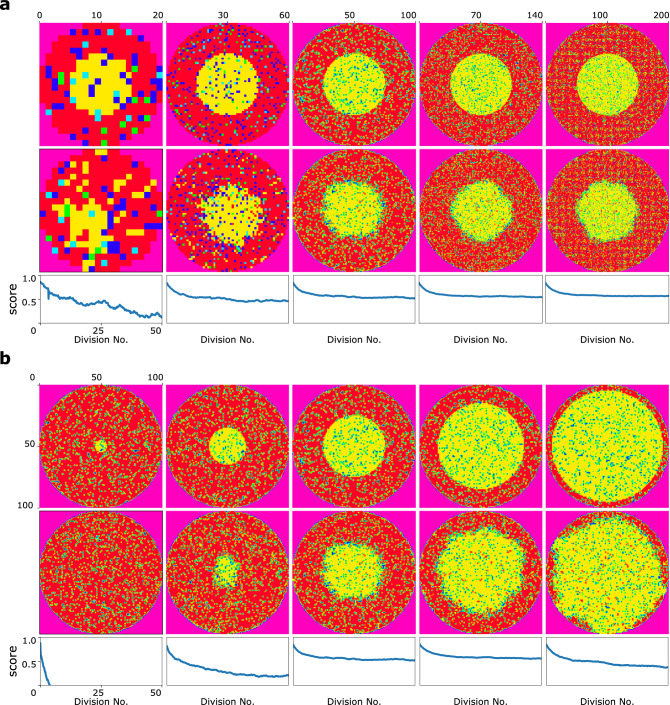
The system behaviour for different simulations. The first, and second rows represent the initial, an final states of the system, respectively. The corresponding score diagram of five mentioned simulations are shown in the last row. (**a**) Five different simulations with the dish radius of 50, but different inner disk size, 5, 15, 25, 35, and 45 from left to right. (**b**) Five different simulations with the dish radius of 50, but different inner disk size, 5, 15, 25, 35, and 45 from left to right.

To show the key role of geometric confinement in maintaining spatial patterns in the population, we repeat the simulation with different sizes of inner disks as well as the whole dish. It is worth-mentioning that from now on all the simulations are done in the presence of signaling molecules. Figure 4a represents the results of five simulations with the same dish radius of 50, but different inner disk size, 5, 15, 25, 35,  and 45 from left to right. The diagrams show that when the number of cells in one territory exceeds, they could easily invade the region occupied by the cells on the opposite side. To study the effect of the whole dish size in maintaining the organized pattern in the population, five simulations are run. Figure 4b demonstrates the results of these five simulations starting with five different dish radius, 10, 30, 50, 70,  and 100, from left to right. In all of these simulations, the inner disk radius is always half of the dish radius. Figure 4b shows that there is a lower bound for the number of cells in the population, for cells to be able to defend their territory, and maintain the spatial pattern in the dish.

### Model behaviour in the face of different population patterns

**Figure 5:**
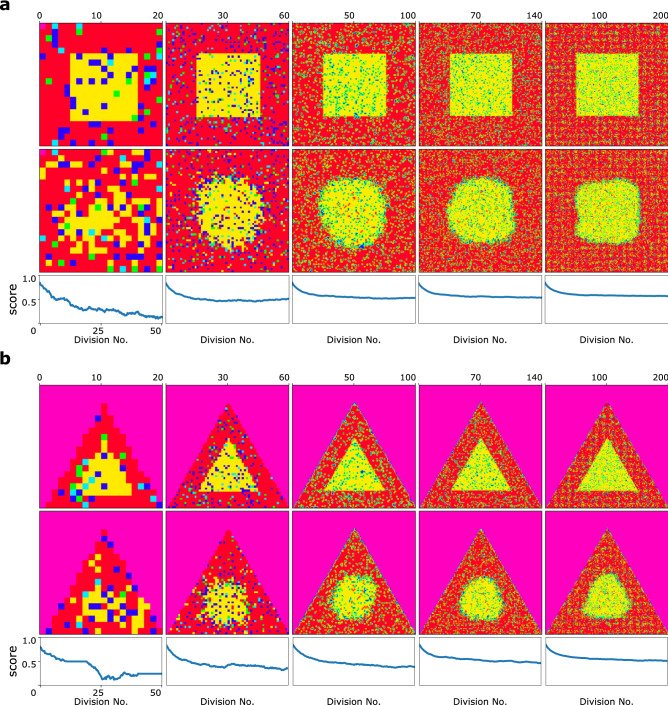
The system behaviour for rectangle shape, and triangle shape dishes. The length of the middle area is always the half of the dish side length. The first, and second rows represent the initial, an final states of the system, respectively. The corresponding score diagram of five mentioned simulations are shown in the last row. (**a**) Five different simulations starting with five rectangle shape dishes of side length, 10, 30, 50, 70, and 100 from left to right. (**b**) Five different simulations starting with five triangle shape dishes of side length, 10, 30, 50, 70, and 100 from left to right.

We simulated the model dynamics on other dish shapes and it is performed with any modification in order to study the effect of changing the geometry of the population. In Fig. 5a,b, the dish is initiated in the shape of a square, and rectangle, respectively. Studying the final results, one could say that there is an inward expansion of *B* cells at the corners of square and rectangular regions. Thus, the model behaves as it is expected based on experiments performed in previous researches^43^. Besides, in Fig. 5 it is clearly shown that by increasing the number of cells in the population, the inner region could be defended more easily by *A* cells.

**Figure 6:**
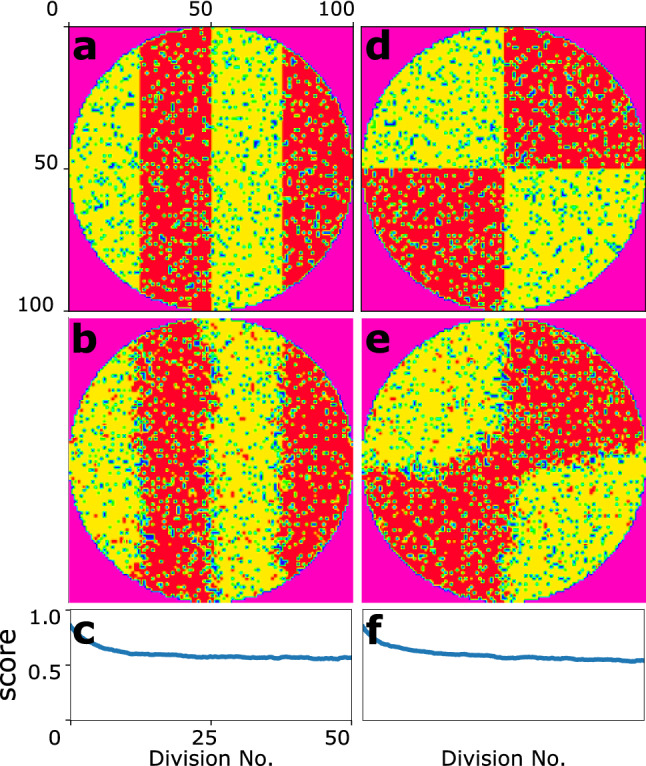
The system behaviour in the face of more complex initial spatial patterns. (**a**,**d**) The initial state of the system. (**b**,**e**) The final states of the system. (**c**,**f**) The corresponding score diagrams.

To challenge the model, we simulated the model in more complex patterns shown in Fig. 6. Here, again one fate has invaded the other fate in the corners. However, the model has been successfully capable of maintaining the initial pattern in the population.

### Model in the face of injuries

**Figure 7:**
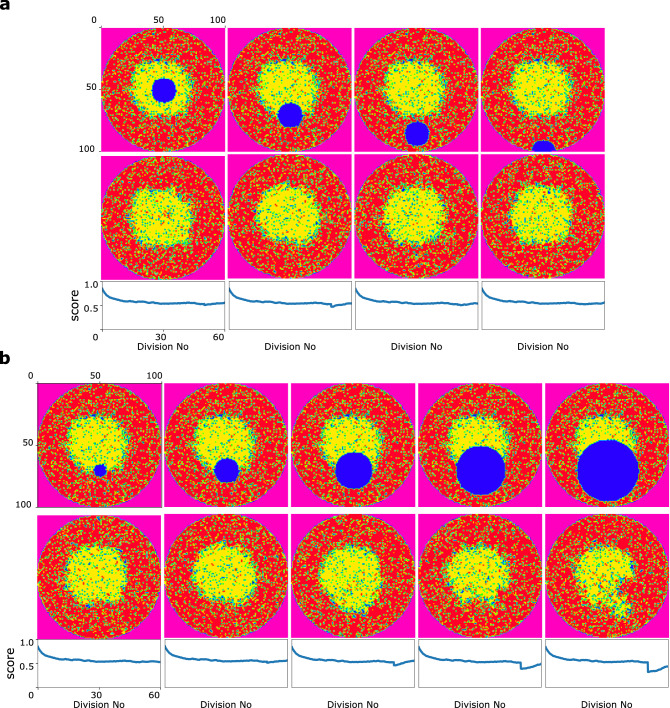
The system behaviour in the face of injuries. The first, and second rows represent the initial, an final states of the system, respectively. The corresponding score diagram of five mentioned simulations are shown in the last row. (**a**) Injuries in different regions in the population. (**b**) Injuries with different regions size.

Studying the results, one could say that our model could properly maintain the organized pattern in the colony. To challenge the model, even more, a new experiment has been designed. In this experiment, in a state in which all progenitor cells have gone through 50 divisions on average(right-hand plot in the second row of Fig. 3), the cells in a part of the dish are diminished at once. This sudden cells death, representing an unexpected injury in the tissue, could happen in different areas of the dish as shown in the first row of Fig. 7a, and also with different sizes, shown in the first row of Fig. 7b. Then we let the system to be updated to the point at which all progenitor cells have gone through 60 divisions, on average (the second row of Fig. 7a,b). Studying the system behavior at this point, it is clear that the model could retrieve the initial pattern appropriately. However, one could say that the loss of cells in the boundaries of regions with different phenotypes is more challenging. Besides, as it is biologically expected the smaller size of the injury region, the more easily the system could recover.

## Discussion

An organism’s life depends on the precise functionality of its organs. On the other hand, the organs’ proper functionality could not be achieved without the proper proportional tissue structure. Needless to say that there is a long way ahead to introduce a comprehensive model which controls the stem cell’s decisions (stem cells as the main source of producing cells) to generate and also maintain the proper structure in the tissue. However, in this project, we took a step toward this tempting big picture and introduced a simple model which could properly maintain the pattern in the tissue.

Here, by focusing on the presence of stochasticity in all levels of cell activities, we define a regulatory switch as a mechanism to maintain both proliferation/differentiation balance and the spatial pattern in a hypothetical normal adult tissue. In the presence of progenitor cells an enormous number of specialized cells (to fulfill the tissue functionality) could be produced with a low rate of stem-cell division. It is pretty safe to conclude that it lowers the risk of mutation accumulation in stem cells!^58^. Besides, progenitor cells with the capability of reversible transfer between states destined for self-renewal or differentiation may provide a surprising degree of flexibility, with recovering stem cell population in the case of injury^60^. Therefore in the most simple model, it is assumed that this hypothetical developing tissue consists of progenitor cells other than stem cells and two differentiated cell types. As expected, stem cells could self-renew and differentiate to the intermediate progenitor cells, while progenitor cells could give birth to progenitor cells and two specialized cell types needed for the functionality of our hypothetical tissue.

The model is described in Eqs. (1), (2), (4), (5), in details. The computation in the rest of “Mathematical model of the system” proves that the described model could hit the homeostatic state under some reachable conditions. In other words, it is proven that starting with any initial condition, the desired proportion of specialized cells to satisfy the tissue functionality and stem, and progenitor cells as the main sources in the tissue, is reassuring. Besides, it shows that this condition could be maintained in a steady state, which is needed in any normal living tissue to survive^59^. Moreover, it reflects the flexibility of our model to describe any desired tissue with a different proportion of differentiated cells.

Two sets of ordinary differential equations are defined to describe the internal regulatory mechanism of the stem (Eq. 26), and progenitor cells (Eq. 27). As discussed before, although the regulatory networks could provide proliferation/differentiation balance and population heterogeneity and, as a result, reach and maintain a homeostasis state, they could not conserve the population structural organization. Therefore, the model is equipped to inter-cellular communication as the third issue required in order to maintain a spatial pattern. The results present evidence for the model’s capability to maintain the organized spatial pattern in the population (Fig. 4a). The results also demonstrate that when one of the regions is much smaller than the other, the dominant cells could capture the territory of cells in the minority (the first and last plots in the second row of Fig. 4a,b). It is completely acceptable since as shown in Eq. (29), signaling molecules $S_1$, and $S_2$ with symmetric mutual inhibition effects protect their territory. Therefore, It is necessary to break the symmetry in Eq. (29) to have a population containing regions with so much difference in population number.

Here, studying different population sizes, consistent with results in previous studies^42,43^ the results suggest that dish size could influence maintaining spatial pattern in the population (Fig. 4b). Specifically, the smaller number of specialized cells in a dish, the fewer number of signaling molecules to defend their corresponding territory.

Importantly, we applied the model without any modification on other shapes (Figs. 5,6) to study the effect of changing the geometry of the colony. The fascinating outcome is that the model could easily maintain the spatial pattern in the population initiated in non-circular and even complex structures. However, an inward expansion of cells is observed at the sharp corners, which is in agreement with previous computational, and experimental predictions in previous studies^43^.

The death of cells caused by injury could cause organs failure due to an insufficient supply to fully restore tissue function. Therefore, as injuries are always a concern, shoulder to shoulder with the maintenance of the desired proportion of specialized cells and ordered spatial pattern, injury-induced repairability is demanded in the tissue^74^. Consequently, we computationally investigated the capability of our model to retrieve the initial pattern in the case of injuries. The results in Fig. 7 show that our model could successfully and without any further modifications restore the initial pattern, which is equivalent to restoring the tissue functionality.

The last but not least point is about the score diagrams shown in the last row of Figs.  4, 5, and 6. The scoring algorithm is designed to evaluate the results in numbers by representing how much the population spatial pattern differs from its initial pattern through 50 divisions. When the number of cells in two territories is both sufficient and in balance, the diagram decreases at the beginning, and at some point, it will get saturated. It clearly verifies that although the territories could not be kept in their exact initial pattern, the system reaches the point with the similarity score of $60\%$, and after that, the structural pattern and the number of different cells will not change anymore. It confirms the stability of the model. Here, a fundamental question could strike minds: what is the reason for having crooked boundaries instead of initial proper ones at the end of the simulations? The obvious answer is stochasticity. The stochasticity could be easily interpreted as a threat to the tissue functionality, which ties in with the tissue organized spatial pattern. On the other hand, stochasticity is the exact factor that brings demanded vital diversity out of the same genotypes in the tissue, the item, which facilitates the tissue functionality. These contradictory roles of stochasticity lead to one of the fascinating outcomes of this project: life is certainly indebted to a controlled amount of uncertainty.

In this model, by emphasizing the prominent role of stochasticity^5,7,13^ in the developed adult tissue, we could maintain the spatial pattern to fulfill the tissue functionality other than the homeostasis state to have a cycling adult tissue^7,60^. We could even maintain more complex patterns and the results agreed with previous studies^43^. Besides, it has been mathematically proved that the introduced system could reach a steady state in all cases under some conditions. While injuries are always possible to happen^74^, with the presence of progenitor cells, we also demonstrate that our model could retrieve the right demanded proportion of different cells in their right position in the case of injuries. To summarize, we introduced a mechanism, by orchestrating the cell decision-making switch and in the presence of intercellular interactions could maintain the population’s overall behavior with no need for any external factors.

Although it is vital to maintain the organized structural pattern in the tissue for the proper functionality, in a comprehensive model, it is also important to probe the stem cells’ strategies to organize the specialized cells to a desired pattern in the colony. Thus, it could be interesting to investigate if our model could initiate pattern formation in the population and study the factors that could affect this process in future work. It could be a promising step toward the big picture of this project: generating organoids starting from stem cells.

## Data Availability

The software used to run all simulations was Python. The scripts and the data that support the findings of this study are available from the corresponding author upon request.
